# Exploring Long Covid Prevalence and Patient Uncertainty by Sociodemographic Characteristics Using GP Patient Survey Data

**DOI:** 10.1111/hex.70202

**Published:** 2025-03-17

**Authors:** Mirembe Woodrow, Nida Ziauddeen, Dianna Smith, Nisreen A. Alwan

**Affiliations:** ^1^ School of Primary Care, Population Sciences and Medical Education, Faculty of Medicine University of Southampton Southampton UK; ^2^ NIHR Applied Research Collaboration Wessex Southampton UK; ^3^ School of Geography and Environment, Faculty of Environmental and Life Sciences The University of Southampton Southampton UK; ^4^ University Hospital Southampton NHS Foundation Trust Southampton UK

**Keywords:** inequality, Long Covid, prevalence, primary care, sociodemographic characteristics

## Abstract

**Background:**

The high global burden of Long Covid (LC) has significant implications for population well‐being, health care, social care and national economies.

**Aim:**

To explore associations between patient sociodemographic and health characteristics with two outcomes: having LC and expressing uncertainty about having LC, as described by general practice (GP) survey respondents.

**Design and Setting:**

Analysis of GP Patient Survey (England), a random sample of 759,149 patients aged 16 years+ registered with a GP in England (2023).

**Method:**

Multivariable logistic regression modelling comparing those with and without LC, and those who were unsure in relation to patient characteristics.

**Results:**

4.8% of respondents reported having LC, and 9.1% were unsure. Significant adjusted associations indicating higher risk of LC included age (highest odds 35−54 years), sex (females), ethnicity (White Gypsy/Irish Traveller, mixed/multiple ethnic groups), sexual orientation (gay/lesbian or bisexual), living in a deprived area, being a carer or a parent and having a long‐term condition (LTC). Those aged ≤ 25 years, males, non‐binary, heterosexual, not parents or carers, from other White, Indian, Bangladeshi, Chinese, Black or Arab backgrounds, former and current smokers, and with no defined LTC were more likely in adjusted analysis to be unsure about having LC compared to answering ‘yes’.

**Conclusion:**

There is an unequal distribution of LC in England, with the condition being more prevalent in minoritised and disadvantaged groups. There are also high levels of uncertainty about having LC. Improved awareness is needed amongst the general population and health care professionals to ensure those most vulnerable in society are identified and provided with care and support.

**Patient or Public Contribution:**

The analysis builds on previous studies co‐created with people with lived experience. A public contributor advised on discussions on dissemination towards optimal impact of this study's findings. Study findings will inform the next phases of the research in which the research questions and design will be co‐created with public partners.

**How This Fits in:**

In England there is high prevalence of Long Covid, a COVID‐19 infection‐induced chronic condition that can limit daily activities significantly. The burden of ill health from Long Covid is unequal, with minoritised groups experiencing higher prevalence. This study adds further evidence of inequality in the prevalence of Long Covid, but also reveals that there are more people who are unsure whether they have Long Covid than those who are confident they have it, with certain groups that are already disadvantaged being more likely to be uncertain if they have the condition. Findings underline a need for greater awareness of Long Covid amongst the public and health care professionals, and for diagnosis, treatment and support to be better distributed according to need.

## 1 Introduction

In the UK, the Office for National Statistics estimated in March 2024 that 2 million adults and children experience Long Covid (LC) (3.3% of the population), with 69% and 41% experiencing it for at least 1 and 2 years respectively and 19% reporting that LC limits their ability to undertake day to day activities ‘a lot’ [1]. There is also evidence of inequalities in LC prevalence, with higher prevalence associated with being female [2, 3, 4, 5, 6, 7, 8, 9], of older or middle age [2, 3, 4, 6, 7], having a higher body mass index (BMI) [2, 3, 5, 7, 8], smoking [3, 5], and belonging to an ethnic minority group [5, 8]. Higher prevalence is also associated with greater deprivation [3, 4, 5, 9]. There is also unequal impact of the condition on people's lives [10, 11, 12] and inequitable support [13, 14, 15] for the condition. More research is needed to understand the extent of this inequality and the action needed to address it.

As a relatively novel condition, knowledge about LC is still in its infancy amongst researchers and health care professionals but there is even more lack of awareness, exacerbated by barriers and stigma, in the general population leaving unwell people unsure if they have LC [16, 17]. This high and unequal burden of illness from LC has significant implications for society in terms of the infrastructure of accessible diagnosis, support and treatment needed for people with LC.

The General Practice Patient Survey (GPPS) is an annual survey of GP‐registered people aged 16 years+ in England, administered by Ipsos on behalf of NHS England [18]. Established in 2007, it asks respondents about their experience of their local GP, other local NHS services and respondents' general health. In 2022 a new question was added that asked whether respondents had LC.

The aims of this study were to (1) explore prevalence of LC, (2) examine its potential sociodemographic and health characteristics and (3) explore factors associated with being unsure about having LC.

## 2 Methods

### 2.1 Sampling

Each year in January the GPPS is issued by post to a random sample of GP‐registered patients in England. It can be completed on paper, online or using a dedicated telephone helpline. Invitations and reminders are also issued by text message and the online survey is available in 14 languages and British Sign Language. Paper copies can be requested in Braille and large print. Data is grouped by geographical and health system factors including general practice (GP), Primary Care Network (PCN), Integrated Care System, Local Authority and NHS Region. Indices of deprivation are also available according to patient postcode. Further technical detail is available online [19].

The survey is issued to approximately 2.5 million registered patients and aims to ensure a minimum number of responses per practice and PCN and a set confidence interval for each practice. A proportionately stratified (by age, gender and postcode) unclustered sample is drawn for each practice [19]. Patient identifiable data is not collected. It is a standalone self‐reported survey that is not linked to GP patient records. The questionnaire was last significantly revised in 2021 (available online) [20]. More detail about the survey is available at https://gp-patient.co.uk/FAQ.

### 2.2 Design

This is an analysis of cross‐sectional survey data using the GPPS data set for 2023. Permission to access and use the individual patient level survey data from 2023 was requested and granted by agreement between NHS England and the University of Southampton. Ethical approval was granted by the University of Southampton Faculty of Medicine Ethics Committee (No.82369).

### 2.3 Variables

The outcome was the response to the question “Would you describe yourself as having ‘Long Covid’, that is, you are still experiencing symptoms more than 12 weeks after you first had COVID‐19, that are not explained by something else?”. Respondents could select ‘yes’, ‘no’, ‘not sure’ or ‘prefer not to say’.

All exposure variables used in the analysis (listed in Supporting Information S1: Tables 1 and 2) were categorical and used as collected with the exception of smoking status. Smoking status was recorded as never, former, occasional and regular smoker and was condensed to never, former and current smoker (regular and occasional) for analysis. A new variable was created to identify respondents with at least one defined long‐term condition (LTC) (other than LC). The question that asked if respondents had any LTCs could not be included as respondents with LC could have counted their LC as a LTC (the LTC variable could include the LC outcome).

### 2.4 Analysis

Data was analysed using Stata v17 [21]. Descriptive statistics, frequencies and proportions were calculated followed by univariable and multivariable logistic regression. The primary analysis compared those answering ‘yes’ and ‘no’ to the LC question, excluding those answering unsure or ‘prefer not to say’. The same analysis comparing those answering ‘not sure’ and ‘no’ was also undertaken, as well as comparing ‘not sure’ and ‘yes’. We conducted the latter analysis to understand the factors associated with uncertainty about having the health condition which may act as a barrier to seeking care and support.

We conducted multivariable analysis to assess the independent effects of variables given they can potentially confound each other. The multivariable analysis included age, sex, sexual orientation, parental/guardian status, carer status, ethnicity, patient's area of residence index of multiple deprivation (IMD) quintile (a combined index of relative deprivation based on data in seven domains) [22], religion, smoking status and having at least one defined LTC (other than LC). Work status was included as an adjustment in the analysis comparing ‘yes’ and ‘not sure’ but not included when comparing ‘yes’ and ‘no’ (since work status could have been impacted by having LC).

## 3 Results

The survey was completed by 759,149 people (28.6% response rate).

**Figure 1:**
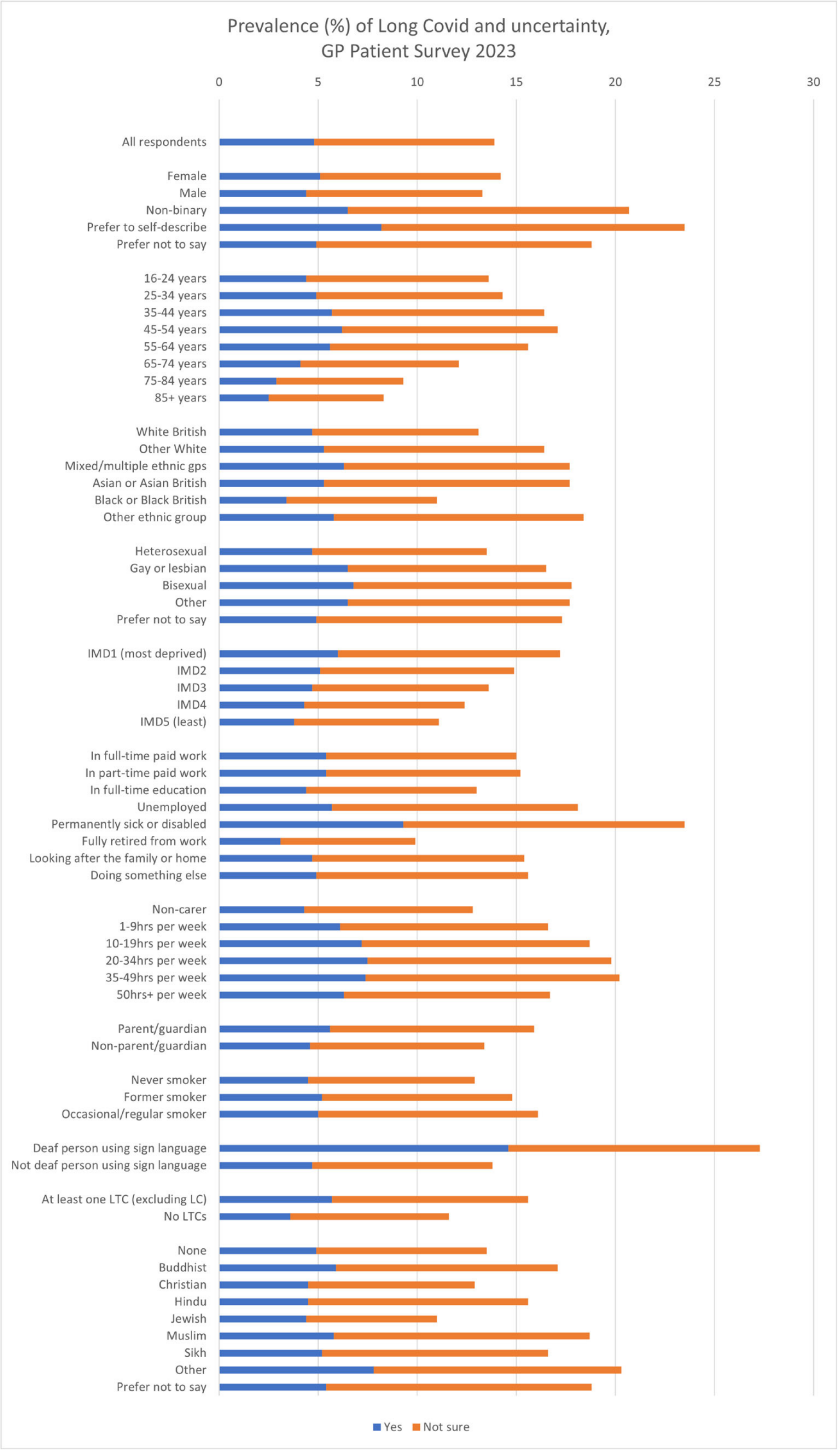
Prevalence (%) of Long Covid and uncertainty, GP Patient Survey 2023.

Figure 1 shows the percentage of respondents answering yes to having LC and answering ‘not sure’. Supporting Information S1: Table 1 additionally describes those answering ‘no’ and ‘prefer not to say’ as well as frequencies.

*Respondents answering yes to having LC (compared with those who answered ‘No’)*

**Figure 2:**
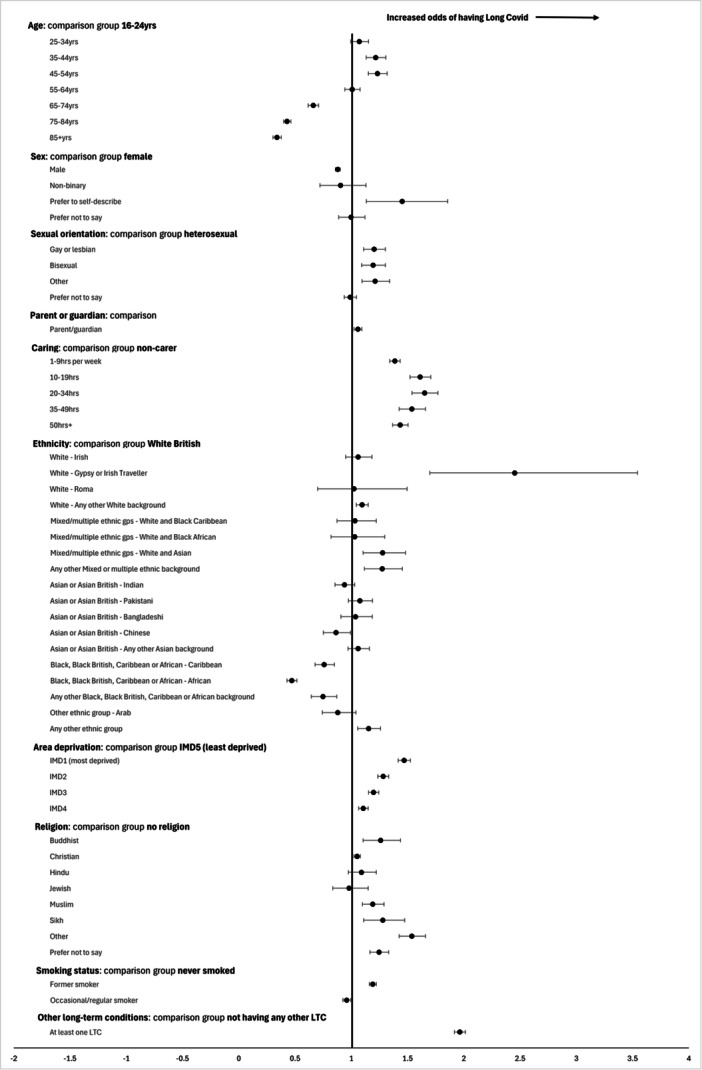
Forest plot of adjusted odds* of having Long Covid compared to not having Long Covid (yes vs no), General Practice Patient Survey data 2023. *All odds ratios are adjusted for all factors in the plot.

Figure 2 shows a forest plot of the odds of having LC compared to not having LC.

The percentage of respondents answering yes to having LC was 4.8% (*n* = 35,445) (Supporting Information S1: Table 1). The highest prevalence of LC was in the North West region (5.5%) and lowest in the South West (3.9%). Nine percent of people who described themselves as permanently sick or disabled had LC. Compared to being in full‐time work, people who were students, retired, looking after the family or home, or ‘doing something else’ were less likely to have LC (Supporting Information S1: Table 2). People who were unemployed or permanently sick or disabled were more likely to have LC (unadjusted 1.12, 95% CI 1.06−1.19; 1.92, 95% CI 1.84−2.00 respectively).

Of those who answered yes to having LC, 59.9% (*n* = 20,973) were female. Males were less likely to answer yes compared to females (adjusted OR 0.88, 95% CI 0.86−0.90) (Supporting Information S1: Table 2). 60.7% of those with LC were aged between 35 and 64 years. Compared to those aged 16−24 years, having LC was significantly less common in those ≥ 65 years and more common in those aged 25−64 years even after adjustment for other characteristics. The prevalence of LC significantly increased as area deprivation increased, with living in the most deprived area quintile associated with 47% higher odds compared to the least (adjusted OR 1.47, 95% CI 1.42−1.53).

People who were gay/lesbian, bisexual or ‘other’ sexual orientations had significantly higher prevalence compared to heterosexuals (Supporting Information S1: Table 2). Parents were more likely to have LC compared to non‐parents, as were carers in all caring hours categories compared to non‐carers. Compared to White British ethnicity, significantly higher adjusted odds of having LC were seen in White Gypsy or Irish Traveller groups, any other White background, White and Asian and ‘other’ mixed or multiple ethnic groups, and ‘any other ethnic group’. All three Black as well as Chinese ethnic groups were less likely to report having LC. Compared to having no religion, adjusted ORs of having LC were higher for Buddhist, Christian, Muslim, Sikh and ‘other’ religions.

The prevalence of LC amongst current regular smokers was 4.8% (Supporting Information S1: Table 1) and the proportion of people with LC who were regular or occasional smokers was 12.2% compared to 11.2% smokers among those with no LC. Compared to people who had never smoked, the adjusted OR of having LC in occasional/regular smokers was 0.96 (95% CI 0.93−1.00) and for former smokers was 1.19 (95% CI 1.16−1.22) (Supporting Information S1: Table 2).

There was particularly high prevalence of LC amongst people who were deaf and using sign language (14.6%), and those who had Alzheimer's/dementia (9.2%), autism (8.8%), a breathing condition (9.0%), a mental health condition (9.2%) or another LTC not listed (7.8%) (Supporting Information S1: Table 1). People with at least one defined LTC (which was not LC) had higher odds of having LC (adjusted OR 1.97, 95% CI 1.92−2.01) (Supporting Information S1: Table 2).

*Respondents who were ‘not sure’ if they would describe themselves as having LC (compared with those who answered ‘Yes’)*

**Figure 3:**
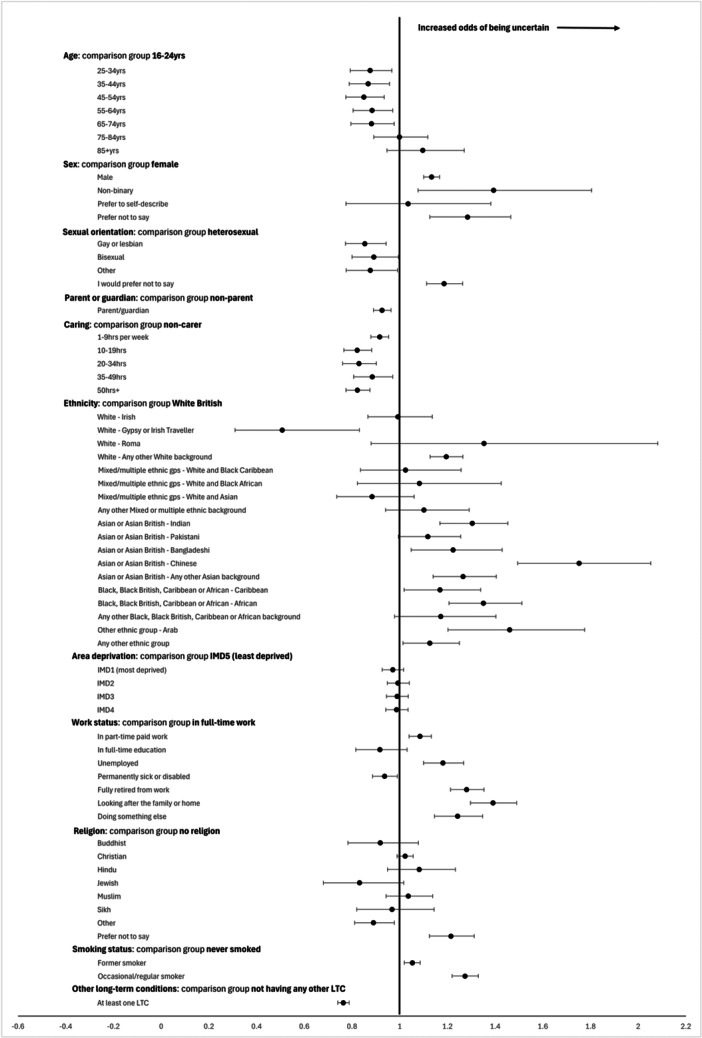
Forest plot of adjusted odds* of being uncertain about having Long Covid compared to having Long Covid (not sure vs. yes), General Practice Patient Survey data 2023. *All odds ratios are adjusted for all factors in the plot.

Figure 3 shows a forest plot of the odds of being uncertain about having LC compared to having LC.

Those aged 25−74 years were significantly less likely to say they were unsure about having LC (compared to those who answered ‘yes’ to having LC) than those aged 16−24 years (Supporting Information S1: Table 3). Males and non‐binary people were significantly more likely to be unsure compared to females. Gay/lesbian and ‘Other’ sexual orientations were significantly less likely to be unsure compared to heterosexuals. Parents were less likely to be unsure compared to non‐parents (adjusted OR 0.93, 95% CI 0.89−0.96), and carers less likely to be unsure compared to non‐carers.

White Gypsy or Irish Traveller were significantly less likely to be unsure that they had LC (compared to those who answered ‘yes’ to having LC) than White British ethnicity. ‘Any other White background’, Indian, Bangladeshi, Chinese, ‘Any other Asian background’, Black Caribbean, Black African, Arab and ‘Any other ethnic group’ were significantly more likely to be unsure compared to White British (Supporting Information S1: Table 3). There was no significant difference by level of area deprivation (IMD quintile) between those who were unsure and those had LC or between those with a religion and those without.

Unemployment, working part‐time, being retired, looking after the family or home, or ‘doing something else’ were positively linked to not being sure as opposed to saying ‘yes’ to having LC, compared with people working full‐time. People who were permanently sick or disabled were less likely to be unsure (Supporting Information S1: Table 3).

Compared with those who answered ‘yes’ to having LC, former or current smokers were significantly more likely to be unsure than people who never smoked. People with at least one defined LTC were less likely to be unsure compared to people without (Supporting Information S1: Table 3).

Results comparing responses of those answering ‘not sure’ and ‘no’ are included in Supporting Information S1: Table 4.

## 4 Discussion

### 4.1 Summary

Analysis of this national GP survey data found that 4.8% of respondents had LC while 9.1% were unsure whether they had it or not. This is a high burden of disease with potential for significant impact on individuals, their families, the economy and wider society. LC is unequally distributed in England, with the condition being more prevalent in minoritised and disadvantaged groups. This study adds new evidence that there are more people who are unsure whether they have LC than those who are confident they have it, again some from more vulnerable groups.

### 4.2 Comparison With Existing Literature

The 4.8% prevalence estimate is similar to that found in other surveys in the UK [1], and latest data from the GP Patient Survey (2024) indicates 5.0% prevalence with 9.0% unsure [18]. Many of this study's findings support existing research on risk factors for LC, for example lower odds of having LC in males compared to females [2, 3, 4, 5, 6, 7, 8, 9], higher odds in those of working age [2, 3, 4, 6, 7], the strong trend of increasing odds of LC with increasing deprivation [1, 3, 4, 5, 9] and higher odds for people who are unemployed [1], and who have existing health conditions [2, 5, 7]. UK studies have also found higher prevalence of LC in particular ethnic minority groups [1, 3, 5, 8] but findings have not been consistent. Lower odds were also found for people of Black ethnicity, something that has been found in other UK studies [1, 7]. Higher prevalence in people from sexual orientation minorities has also been identified in one US study [23].

In this study, there were higher odds of having LC for people who are parents and carers. This could be explained by disproportionate exposure to SARS‐CoV‐2 through schools and caring roles, or greater awareness of LC in these groups. The latter is supported by the reduced odds found in this study of being unsure about having LC in those groups. The impact of LC symptoms could be more noticeable due to caring roles, or the likelihood of being able to rest during the acute COVID‐19 phase may have been limited (a potential factor in some LC cases [10]). There were also higher odds for people of particular faiths, even after adjusting for ethnicity, deprivation, age, gender and other potential confounding factors. However, this still may be explained by residual confounding. Odds of having LC were higher in former smokers but lower for current smokers, which is contrary to studies where smoking has been identified as a risk factor [3, 5]. However, reviews of this area recognise the variation in findings about the role of smoking in LC [24].

This study adds new evidence about the lack of certainty people feel about whether or not they have LC. Groups who were less confident about having LC included males and non‐binary people. In men this could be explained by a tendency to consult less [25] or delay health‐related help‐seeking behaviour [26]. Also less confident were people from ‘Any other white background’, Indian, Bangladeshi, Chinese, ‘Any other Asian background’, Black Caribbean, Black African, Arab and ‘Any other ethnic group’, those who were working part‐time, unemployed, retired, or staying at home and smokers. Those who were less likely to be unsure about having LC included those aged 24−74 years, those who were not heterosexual, parents/guardians and carers, White Gypsy/Irish Traveller, people who were sick or disabled and people with at least one defined LTC. People with LTCs in addition to LC may be less unsure because they are clear about the difference between the symptoms arising from their other LTCs and their LC symptoms. It is also possible that people with other LTCs are more used to navigating the health system, or have increased health literacy due to their LTC history. Those under the age of 25 years were less confident about having LC. This could explain why most research about LC includes older people: most uses self‐identification with LC as study inclusion strategy. It could also be the case that there is wider associated stigma with having LC in younger people [17].

### 4.3 Strengths and Limitations

A key strength of this study is its large and national survey sample size. The GP Patient Survey is a very useful source of information on LC in England, particularly in light of the discontinuation of other national surveys. The demographic information about participants is particularly rich, for example it includes information about the travelling community which can often be difficult to source.

Limitations include lack of data about severity (e.g., hospitalisation) or symptoms of LC, SARS‐CoV‐2 variant, timing of onset and respondents' COVID‐19 vaccination status. Having this information would provide more context. Additionally, interpreting survey responses about LTCs with respect to LC is problematic. LC is a LTC but 21.9% of people who described themselves as having LC said they did not have any LTCs. To take account of this the multivariable models in this study included a variable constructed from named LTCs as reported by respondents rather than the answer to the question ‘any other LTC’ to eliminate potentially including the LC outcome. A further limitation is that we cannot role‐reverse causality for some of the variables such as work status, hence this variable was not included in the multivariable analysis.

### 4.4 Implications for Research and/or Practice

Given the high, unequal burden of LC in the global population and its impact on everyday lives of individuals and the functioning of society, it is important to understand the drivers for this inequality and for the uncertainty around LC to enable people to get the right support. Evidence on access to LC treatment and support indicates there is inequity on many levels. Exploring data from NHS England about access to COVID‐19 Post‐Covid Assessment Services [27] during the period of the GPPS survey 2023 reveals large differences in waiting times between regions, and those differences do not correspond to differences in regional prevalence of LC.

Levels of uncertainty may indicate a lack of awareness of LC symptoms or confusion, or there may be an overlap in symptoms with other conditions. People who are unsure may have less severe LC symptoms, or may have symptoms that are improving or are currently in remission, and so are no longer sure if they still have LC. They may also be waiting for a LC diagnosis, or unsure if they had previously had a COVID‐19 infection. A lack of uncertainty in particular groups may be because a diagnosis has already been secured, indicating that some groups may be more likely than others to be successfully diagnosed, or it could be related to levels of health literacy and self‐advocacy. It may also be the case that people who are sure they have LC are likely to be most severely impacted. Stigma and self‐doubt can shape self‐identification with LC [16, 17, 28, 29, 30] which could be discouraging people from coming forward for diagnosis and support, and there is emerging evidence that intersectional identity may compound LC stigma leading to worse outcomes for marginalised groups [16]. Added to this, LC is itself an uncertain illness, with symptoms and impact varying between people and changing over time, and coupled with a lack of clear pathology or biomarkers, this can result in difficulty with diagnosis and may result in hermeneutical (interpretative) injustice [16]. This is a type of epistemic injustice in LC, with people's experiences often being questioned or viewed as unreliable. Another type of epistemic injustice is testimonial injustice: the degree by which a person is not believed about their own story depends on their identity [31]. This has led to calls for the lived experience of people with LC to be consistently acknowledged and given validation [29]. Our study has provided a useful estimate to quantify the uncertainty brought about by these numerous factors.

With involvement of PPIE partners, we plan further analysis using this survey, looking at the experience of support in primary care for people with LC. We will also link survey data with place‐based data to consider multi‐level factors affecting variation in prevalence and uncertainty. Together with PPIE, the current analysis will be crucial in informing our planned qualitative work to understand the mechanisms that drive inequality in LC prevalence, impact, support and wider societal effects.

## 5 Conclusion

This clearly demonstrable health inequality lends weight to the argument for diagnosis, treatment and support to be better distributed according to need [32], to avoid LC becoming another factor contributing to the widening health gap between different groups in society. The evidence suggests that LC prevalence is not declining, and understanding this novel condition and its impacts requires much greater investment in research. Learning from and comparisons with other similar LTCs such as myalgic encephalomyelitis (Chronic Fatigue Syndrome or ME/CFS) may also be helpful in adding to the body of knowledge.

Improved awareness about the condition amongst the general population is required, and in addition effort should be made to target campaigns towards groups in which LC is more prevalent. The importance of partnering with PPIE contributors in developing awareness campaigns amongst the public and health care professionals cannot be underestimated. Similarly, co‐produced training for health care professionals about barriers and stigma associated with LC is required to ensure people with LC can be identified and provided with the right treatment and support.

## Author Contributions

**Mirembe Woodrow:** conceptualisation, investigation, writing – original draft, methodology, writing – review and editing, formal analysis. **Nida Ziauddeen:** conceptualisation, methodology, writing – review and editing, supervision, validation. **Dianna Smith:** conceptualisation, methodology, writing – review and editing – supervision. **Nisreen A. Alwan:** conceptualisation – writing – review and editing – methodology – supervision.

## Ethics Statement

Ethical approval was granted by the University of Southampton Faculty of Medicine Ethics Committee (No.82369).

## Conflicts of Interest

N.A.A. has lived experience of Long Covid, reports grants from National Institute for Health and Care Research for Long Covid research (STIMULATE‐ICP and Hi‐COVE studies), and is a Long Covid Support Charity Scientific Advisor. The other authors declare no conflicts of interest.

## Supporting information

Supporting information.

## Data Availability

G.P. Patient Survey data is available at G.P. practice‐level via https://gppatient.co.uk/analysistool. Requests for GPPS patient‐level data sets can be made directly to the survey team whose details can be found here https://gp-patient.co.uk/confidentiality.
