# Supplementary material for: Exploring Long Covid Prevalence and Patient Uncertainty by Sociodemographic Characteristics Using GP Patient Survey Data
Source: Health Expect. 2025 Mar 17;28(2):e70202. doi: 10.1111/hex.70202 (PMC11913530; doi:10.1111/hex.70202)
Supplement: Supplementary file 1 — Supporting information. [file HEX-28-e70202-s001.docx]

**Supplementary material**

Contents

[Table S1: Characteristics of the study population by Long Covid status as reported by survey participants (n=759,149) 1](#_Toc185255494)

[Table S2: Odds of answering yes to having Long Covid compared to not having Long Covid (yes vs no) using General Practice Patient Survey data (2023) 5](#_Toc185255495)

[Table S3: Odds of being uncertain about having Long Covid compared to reporting having Long Covid (not sure vs yes) using General Practice Patient Survey data (2023) 9](#_Toc185255496)

[Table S4: Odds of being uncertain about having Long Covid compared to not having Long Covid (not sure vs no) using General Practice Patient Survey data (2023) 13](#_Toc185255497)

## Table S1: Characteristics of the study population by Long Covid status as reported by survey participants (n=759,149)

|  | **Yes** | **No** | **Not sure** | **Prefer not to say** |
| --- | --- | --- | --- | --- |
|  | n (%) | n (%) | n (%) | n (%) |
| **All respondents** | | | | |
|  | 35445 (4.8) | 632748 (85.3) | 67257 (9.1) | 5991 (0.8) |
| **Commissioning region** | | | | |
| East | 3379 (4.3) | 68103 (86.7) | 6625 (8.4) | 486 (0.6) |
| London | 6158 (4.7) | 110489 (84.2) | 12859 (9.8) | 1732 (1.3) |
| Midlands | 7450 (5.1) | 124690 (84.6) | 13922 (9.5) | 1319 (0.9) |
| North East | 5687 (5.1) | 94826 (84.9) | 10408 (9.3) | 800 (0.7) |
| North West | 6095 (5.5) | 93015 (84.0) | 10781 (9.7) | 890 (0.8) |
| South East | 4111 (4.2) | 84642 (87.3) | 7728 (8.0) | 519 (0.5) |
| South West | 2565 (3.9) | 56983 (88.0) | 4934 (7.6) | 245 (0.4) |
| **Sex** | | | | |
| Female | 20973 (5.1) | 353888 (85.2) | 37635 (9.1) | 2649 (0.6) |
| Male | 13444 (4.4) | 263956 (86.1) | 27246 (8.9) | 2062 (0.7) |
| Non-binary | 102 (6.5) | 1214 (76.7) | 224 (14.2) | 42 (2.7) |
| Prefer to self-describe | 82 (8.2) | 693 (69.3) | 153 (15.3) | 72 (7.2) |
| Prefer not to say | 391 (4.9) | 5502 (69.4) | 1104 (13.9) | 937 (11.8) |
| **Age** | | | | |
| 16-24 years | 1148 (4.4) | 22183 (84.9) | 2391 (9.2) | 403 (1.5) |
| 25-34 years | 2759 (4.9) | 47858 (84.4) | 5327 (9.4) | 745 (1.3) |
| 35-44 years | 5039 (5.7) | 73225 (82.3) | 9535 (10.7) | 1171 (1.3) |
| 45-54 years | 7313 (6.2) | 95929 (81.8) | 12793 (10.9) | 1184 (1.0) |
| 55-64 years | 9027 (5.6) | 135435 (83.8) | 16107 (10.0) | 1079 (0.7) |
| 65-74 years | 6349 (4.1) | 137287 (87.5) | 12556 (8.0) | 684 (0.4) |
| 75-84 years | 2946 (2.9) | 92496 (90.3) | 6577 (6.4) | 443 (0.4) |
| 85+ years | 664 (2.5) | 24716 (91.2) | 1557 (5.8) | 162 (0.6) |
| **Ethnicity** | | | | |
| White - English, Welsh, Scottish, Northern Irish or British | 25968 (4.7) | 479699 (86.5) | 46529 (8.4) | 2260 (0.4) |
| White – Irish | 362 (4.7) | 6565 (86.0) | 678 (8.9) | 31 (0.4) |
| White - Gypsy or Irish Traveller | 42 (14.6) | 203 (70.5) | 36 (12.5) | 7 (2.4) |
| White - Roma | 33 (5.1) | 495 (76.3) | 87 (13.4) | 34 (5.2) |
| White - Any other White background | 2185 (5.4) | 33154 (81.4) | 4692 (11.5) | 683 (1.7) |
| Mixed or Multiple ethnic groups - White and Black Caribbean | 154 (5.7) | 2214 (82.6) | 279 (10.4) | 35 (1.3) |
| Mixed or Multiple ethnic groups - White and Black African | 87 (5.7) | 1240 (81.6) | 167 (11.0) | 25 (1.7) |
| Mixed or Multiple ethnic groups - White and Asian | 209 (6.4) | 2674 (81.6) | 348 (10.6) | 46 (1.4) |
| Mixed or Multiple ethnic groups - Any other Mixed or multiple ethnic background | 263 (6.8) | 3063 (78.8) | 503 (12.9) | 59 (1.5) |
| Asian or Asian British - Indian | 1319 (4.7) | 23244 (81.9) | 3227 (11.4) | 592 (2.1) |
| Asian or Asian British – Pakistani | 1111 (6.4) | 13483 (77.1) | 2341 (13.4) | 551 (3.2) |
| Asian or Asian British - Bangladeshi | 368 (6.2) | 4522 (76.6) | 851 (14.4) | 159 (2.7) |
| Asian or Asian British - Chinese | 233 (4.0) | 4814 (82.1) | 725 (12.4) | 90 (1.5) |
| Asian or Asian British - Any other Asian background | 707 (5.4) | 10503 (80.1) | 1616 (12.3) | 279 (2.1) |
| Black, Black British, Caribbean or African - Caribbean | 349 (4.3) | 7007 (85.9) | 704 (8.6) | 100 (1.2) |
| Black, Black British, Caribbean or African - African | 523 (2.9) | 16244 (89.2) | 1219 (6.7) | 220 (1.2) |
| Black, Black British, Caribbean or African - Any other Black, Black British, Caribbean or African background | 193 (4.22) | 3902 (85.4) | 418 (9.1) | 58 (1.2) |
| Other ethnic group - Arab | 191 (5.1) | 2982 (79.2) | 492 (13.1) | 102 (2.7) |
| Any other ethnic group | 707 (6.0) | 9106 (77.7) | 1465 (12.5) | 441 (3.8) |
| **Sexual orientation** | | | | |
| Heterosexual | 31093 (4.7) | 568277 (86.0) | 58132 (8.8) | 3206 (0.5) |
| Gay or lesbian | 723 (6.5) | 9267 (83.1) | 1119 (10.0) | 47 (0.4) |
| Bisexual | 609 (6.8) | 7310 (81.2) | 990 (11.0) | 93 (1.0) |
| Other | 500 (6.5) | 6164 (79.8) | 868 (11.2) | 197 (2.6) |
| I would prefer not to say | 1939 (4.9) | 30491 (77.1) | 4916 (12.4) | 2192 (5.5) |
| **Gender identity is the same as the sex registered at birth** | | | | |
| Yes | 34344 (4.8) | 627191 (85.6) | 64753 (9.0) | 4543 (0.6) |
| No | 267 (6.3) | 3289 (77.9) | 534 (12.6) | 134 (3.2) |
| Prefer not to say | 451 (5.3) | 5626 (66.5) | 1244 (14.7) | 1140 (13.5) |
| **Patient area of residence IMD quintile** | | | | |
| 1 (most deprived) | 8917 (6.0) | 121888 (81.4) | 16715 (11.2) | 2207 (1.5) |
| 2 | 7606 (5.1) | 125690 (84.0) | 14726 (9.8) | 1589 (1.1) |
| 3 | 7082 (4.7) | 130237 (85.8) | 13464 (8.9) | 1021 (0.7) |
| 4 | 6414 (4.3) | 129861 (87.2) | 12011 (8.1) | 717 (0.5) |
| 5 (least) | 5408 (3.8) | 124732 (88.5) | 10300 (7.3) | 452 (0.3) |
| **Work status** | | | | |
| In full-time paid work (30 hours or more each week) | 13777 (5.4) | 213406 (84.2) | 24409 (9.6) | 1891 (0.8) |
| In part-time paid work (under 30 hours each week) | 4958 (5.4) | 77225 (84.1) | 8955 (9.8) | 667 (0.7) |
| In full-time education at school, college or university | 657 (4.4) | 12649 (85.7) | 1270 (8.6) | 188 (1.3) |
| Unemployed | 1425 (5.7) | 19703 (79.4) | 3068 (12.4) | 609 (2.5) |
| Permanently sick or disabled | 3222 (9.3) | 25998 (75.4) | 4886 (14.2) | 389 (1.1) |
| Fully retired from work | 7742 (3.1) | 222069 (89.8) | 16725 (6.8) | 873 (0.4) |
| Looking after the family or home | 1527 (4.7) | 26989 (83.3) | 3472 (10.7) | 404 (1.3) |
| Doing something else | 1033 (4.9) | 17284 (81.2) | 2277 (10.7) | 689 (3.2) |
| **Carer** | | | | |
| No | 24598 (4.3) | 492447 (86.4) | 48681 (8.5) | 4502 (0.8) |
| 1-9hrs per week | 4693 (6.1) | 63947 (83.0) | 8121 (10.5) | 331 (0.4) |
| 10-19hrs per week | 1436 (7.2) | 16099 (80.6) | 2296 (11.5) | 150 (0.8) |
| 20-34hrs per week | 986 (7.5) | 10444 (79.0) | 1626 (12.3) | 157 (1.2) |
| 35-49hrs per week | 832 (7.4) | 8786 (78.1) | 1435 (12.8) | 196 (1.7) |
| 50hrs+ per week | 2107 (6.3) | 27570 (82.4) | 3461 (10.4) | 313 (0.9) |
| **Parent or guardian** | | | | |
| Yes | 7760 (5.6) | 114317 (82.9) | 14185 (10.3) | 1683 (1.2) |
| No | 27353 (4.6) | 513023 (85.9) | 52426 (8.8) | 4148 (0.7) |
| **Smoking status** | | | | |
| Never smoker | 18997 (4.5) | 364068 (86.3) | 35260 (8.4) | 3542 (0.8) |
| Former smoker | 11834 (5.2) | 192805 (84.7) | 21851 (9.6) | 1129 (0.5) |
| Occasional/regular smoker | 4302 (5.0) | 70511 (82.5) | 9506 (11.1) | 1154 (1.4) |
| **Deaf person who uses sign language** | | | | |
| Yes | 447 (14.6) | 2075 (67.7) | 389 (12.7) | 154 (5.0) |
| No | 34527 (4.7) | 623399 (85.5) | 65998 (9.1) | 5662 (0.8) |
| **Long term conditions** | | | | |
| Any long-term condition* | 26200 (5.8) | 378567 (83.9) | 44453 (9.9) | 1948 (0.4) |
| Any long-term condition excluding LC (derived variable) | 23443 (5.7) | 343728 (83.9) | 40632 (9.9) | 1690 (0.4) |
| Alzheimer’s disease or other cause of dementia | 545 (9.2) | 4922 (82.8) | 444 (7.5) | 32 (0.5) |
| Arthritis or ongoing problem with back or joints | 10936 (6.4) | 139453 (82.1) | 18915 (11.1) | 622 (0.4) |
| Autism or autism spectrum condition | 528 (8.8) | 4742 (78.7) | 710 (11.8) | 43 (0.7) |
| Blindness or partial sight | 621 (5.3) | 9794 (84.1) | 1162 (10.0) | 64 (0.6) |
| Breathing condition such as asthma or COPD | 7757 (9.0) | 66590 (77.6) | 11132 (13.0) | 390 (0.5) |
| Cancer (diagnosis or treatment in the last 5 years) | 1253 (3.9) | 28620 (88.0) | 2543 (7.8) | 94 (0.3) |
| Deafness or hearing loss | 2981 (5.2) | 48069 (84.6) | 5605 (9.9) | 194 (0.3) |
| Diabetes | 4161 (5.7) | 61316 (83.6) | 7432 (10.1) | 405 (0.6) |
| Heart condition such as angina or atrial fibrillation | 3199 (5.8) | 45937 (83.8) | 5510 (10.1) | 196 (0.4) |
| High blood pressure | 8584 (5.4) | 135479 (84.4) | 15788 (9.8) | 617 (0.4) |
| Kidney or liver disease | 1211 (6.7) | 14477 (80.6) | 2159 (12.0) | 115 (0.6) |
| Learning disability | 625 (7.8) | 6283 (78.5) | 1004 (12.5) | 95 (1.2) |
| Mental health condition | 6332 (9.2) | 52790 (76.6) | 9472 (13.7) | 369 (0.5) |
| Neurological condition such as epilepsy | 922 (6.5) | 11724 (82.9) | 1442 (10.2) | 53 (0.4) |
| Stroke (which affects day-to-day life) | 478 (6.1) | 6455 (82.3) | 856 (10.9) | 51 (0.7) |
| Another long-term condition or disability | 7830 (7.8) | 80496 (80.6) | 11186 (11.2) | 402 (0.4) |
| *No long-term condition* | *7176 (3.1)* | *208447 (88.7)* | *17565 (7.5)* | *1711 (0.7)* |
| **Religion** | | | | |
| None | 10477 (4.9) | 184896 (86.1) | 18564 (8.6) | 868 (0.4) |
| Buddhist | 290 (5.9) | 3959 (81.1) | 545 (11.2) | 85 (1.7) |
| Christian | 18359 (4.5) | 354716 (86.6) | 34526 (8.4) | 2002 (0.5) |
| Hindu | 713 (4.5) | 12980 (82.6) | 1741 (11.1) | 281 (1.8) |
| Jewish | 190 (4.4) | 3793 (88.5) | 281 (6.6) | 23 (0.5) |
| Muslim | 2457 (5.8) | 33111 (78.5) | 5420 (12.9) | 1176 (2.8) |
| Sikh | 377 (5.2) | 5821 (80.5) | 826 (11.4) | 211 (2.9) |
| Other | 883 (7.8) | 8892 (78.4) | 1412 (12.5) | 151 (1.3) |
| Prefer not to say | 1301 (5.4) | 18609 (76.9) | 3232 (13.4) | 1050 (4.3) |

* Presented as reported in the survey, but this may include the LC outcome

## Table S2: Odds of answering yes to having Long Covid compared to not having Long Covid (yes vs no) using General Practice Patient Survey data (2023)

|  | **Unadjusted** | | | | **Adjusted** | | | |
| --- | --- | --- | --- | --- | --- | --- | --- | --- |
|  | Odds ratio | 95% CI  lower | 95% CI  upper | p | Odds ratio | 95% CI  lower | 95% CI  upper | p |
| **Age** | | | | | | | | |
| 16-24yrs | ref |  |  |  | ref |  |  |  |
| 25-34yrs | 1.114 | 1.040 | 1.196 | 0.003 | 1.071 | 0.995 | 1.153 | 0.068 |
| 35-44yrs | 1.330 | 1.245 | 1.420 | <0.0001 | 1.216 | 1.133 | 1.306 | <0.0001 |
| 45-54yrs | 1.473 | 1.382 | 1.570 | <0.0001 | 1.232 | 1.151 | 1.318 | <0.0001 |
| 55-64yrs | 1.288 | 1.209 | 1.372 | <0.0001 | 1.008 | 0.942 | 1.078 | 0.819 |
| 65-74yrs | 0.894 | 0.838 | 0.953 | 0.001 | 0.662 | 0.617 | 0.709 | <0.0001 |
| 75-84yrs | 0.615 | 0.574 | 0.660 | <0.0001 | 0.429 | 0.398 | 0.463 | <0.0001 |
| 85+yrs | 0.519 | 0.471 | 0.572 | <0.0001 | 0.339 | 0.304 | 0.378 | <0.0001 |
| **Sex** | | | | | | | | |
| Female | ref |  |  |  | ref |  |  |  |
| Male | 0.859 | 0.841 | 0.879 | <0.0001 | 0.879 | 0.859 | 0.900 | <0.0001 |
| Non-binary | 1.418 | 1.15 | 1.736 | 0.001 | 0.904 | 0.722 | 1.130 | 0.375 |
| Prefer to self-describe | 1.997 | 1.587 | 2.511 | <0.0001 | 1.451 | 1.134 | 1.856 | 0.003 |
| Prefer not to say | 1.199 | 1.081 | 1.330 | 0.001 | 0.998 | 0.889 | 1.121 | 0.974 |
| **Sexual orientation** | | | | | | | | |
| Heterosexual | ref |  |  |  | ref |  |  |  |
| Gay or lesbian | 1.426 | 1.320 | 1.539 | <0.0001 | 1.203 | 1.110 | 1.303 | <0.0001 |
| Bisexual | 1.523 | 1.401 | 1.655 | <0.0001 | 1.193 | 1.092 | 1.303 | <0.0001 |
| Other | 1.483 | 1.352 | 1.625 | <0.0001 | 1.212 | 1.096 | 1.339 | <0.0001 |
| I would prefer not to say | 1.162 | 1.109 | 1.219 | <0.0001 | 0.990 | 0.937 | 1.046 | 0.714 |
| **Gender identity is the same as the sex registered at birth** | | | | | | | | |
| Yes | ref |  |  |  | NA |  |  |  |
| No | 1.459 | 1.287 | 1.653 | <0.0001 |  |  |  |  |
| Prefer not to say | 1.441 | 1.308 | 1.587 | <0.0001 |  |  |  |  |
| **Parent** | | | | | | | | |
| No | ref |  |  |  | ref |  |  |  |
| Yes | 1.273 | 1.240 | 1.307 | <0.0001 | 1.058 | 1.024 | 1.093 | 0.001 |
| **Carer** | | | | | | | | |
| No | ref |  |  |  |  |  |  |  |
| 1-9hrs per week | 1.469 | 1.423, | 1.517 | <0.0001 | 1.387 | 1.341 | 1.434 | <0.0001 |
| 10-19hrs | 1.786 | 1.689 | 1.888 | <0.0001 | 1.612 | 1.522 | 1.707 | <0.0001 |
| 20-34hrs | 1.890 | 1.768 | 2.020 | <0.0001 | 1.651 | 1.540 | 1.769 | <0.0001 |
| 35-49hrs | 1.896 | 1.764 | 2.038 | <0.0001 | 1.538 | 1.426 | 1.658 | <0.0001 |
| 50hrs+ | 1.530 | 1.461 | 1.602 | <0.0001 | 1.434 | 1.367 | 1.505 | <0.0001 |
| **Ethnicity** | | | | | | | | |
| White - English, Welsh, Scottish, Northern Irish or British | ref |  |  |  | ref |  |  |  |
| White – Irish | 1.019 | 0.916 | 1.133 | 0.735 | 1.060 | 0.949 | 1.183 | 0.302 |
| White - Gypsy or Irish Traveller | 3.822 | 2.741 | 5.329 | <0.0001 | 2.452 | 1.697 | 3.544 | <0.0001 |
| White - Roma | 1.232 | 0.866 | 1.752 | 0.247 | 1.024 | 0.701 | 1.495 | 0.903 |
| White - Any other White background | 1.217 | 1.164 | 1.274 | <0.0001 | 1.096 | 1.044 | 1.150 | <0.0001 |
| Mixed or Multiple ethnic groups - White and Black Caribbean | 1.285 | 1.091 | 1.514 | 0.003 | 1.033 | 0.872 | 1.222 | 0.708 |
| Mixed or Multiple ethnic groups - White and Black African | 1.296 | 1.042 | 1.611 | 0.020 | 1.031 | 0.820 | 1.297 | 0.791 |
| Mixed or Multiple ethnic groups - White and Asian | 1.444 | 1.254 | 1.663 | <0.0001 | 1.278 | 1.104 | 1.481 | 0.001 |
| Mixed or Multiple ethnic groups - Any other Mixed or multiple ethnic background | 1.586 | 1.398 | 1.800 | <0.0001 | 1.274 | 1.116 | 1.454 | <0.0001 |
| Asian or Asian British - Indian | 1.048 | 0.990 | 1.110 | 0.104 | 0.939 | 0.855 | 1.031 | 0.185 |
| Asian or Asian British – Pakistani | 1.522 | 1.430 | 1.620 | <0.0001 | 1.077 | 0.975 | 1.189 | 0.146 |
| Asian or Asian British - Bangladeshi | 1.503 | 1.350 | 1.673 | <0.0001 | 1.037 | 0.907 | 1.186 | 0.590 |
| Asian or Asian British – Chinese | 0.894 | 0.783 | 1.020 | 0.097 | 0.864 | 0.751 | 0.994 | 0.041 |
| Asian or Asian British - Any other Asian background | 1.243 | 1.151 | 1.343 | <0.0001 | 1.061 | 0.971 | 1.160 | 0.189 |
| Black, Black British, Caribbean or African - Caribbean | 0.920 | 0.826 | 1.025 | 0.131 | 0.758 | 0.677 | 0.849 | <0.0001 |
| Black, Black British, Caribbean or African – African | 0.595 | 0.545 | 0.649 | <0.0001 | 0.470 | 0.428 | 0.517 | <0.0001 |
| Black, Black British, Caribbean or African - Any other Black, Black British, Caribbean or African background | 0.914 | 0.790 | 1.056 | 0.223 | 0.748 | 0.644 | 0.870 | <0.0001 |
| Other ethnic group - Arab | 1.183 | 1.022 | 1.370 | 0.025 | 0.879 | 0.743 | 1.041 | 0.136 |
| Any other ethnic group | 1.434 | 1.327 | 1.530 | <0.0001 | 1.154 | 1.057 | 1.260 | 0.001 |
| **Patient IMD quintile** | | | | | | | | |
| 1 (most deprived) | 1.687 | 1.630 | 1.747 | <0.0001 | 1.470 | 1.416 | 1.526 | <0.0001 |
| 2 | 1.396 | 1.347 | 1.446 | <0.0001 | 1.283 | 1.236 | 1.333 | <0.0001 |
| 3 | 1.254 | 1.210 | 1.300 | <0.0001 | 1.198 | 1.154 | 1.244 | <0.0001 |
| 4 | 1.139 | 1.098 | 1.182 | <0.0001 | 1.106 | 1.065 | 1.150 | <0.0001 |
| 5 (least) | ref |  |  |  |  |  |  |  |
| **Work status** | | | | | | | | |
| In full-time paid work (30 hours or more each week) | ref |  |  |  | Not included |  |  |  |
| In part-time paid work (under 30 hours each week) | 0.994 | 0.962 | 1.028 | 0.746 |  |  |  |  |
| In full-time education at school, college or university | 0.805 | 0.742 | 0.872 | <0.0001 |  |  |  |  |
| Unemployed | 1.120 | 1.059 | 1.185 | <0.0001 |  |  |  |  |
| Permanently sick or disabled | 1.920 | 1.844 | 2.000 | <0.0001 |  |  |  |  |
| Fully retired from work | 0.540 | 0.525 | 0.556 | <0.0001 |  |  |  |  |
| Looking after the family or home | 0.876 | 0.830 | 0.925 | <0.0001 |  |  |  |  |
| Doing something else | 0.926 | 0.867 | 0.988 | 0.020 |  |  |  |  |
| **Religion** | | | | | | | | |
| None | ref |  |  |  | ref |  |  |  |
| Buddhist | 1.293 | 1.146 | 1.459 | 0.001 | 1.260 | 1.104 | 1.438 | 0.001 |
| Christian | 0.913 | 0.891 | 0.936 | <0.0001 | 1.051 | 1.024 | 1.080 | <0.0001 |
| Hindu | 0.969 | 0.897 | 1.048 | 0.434 | 1.091 | 0.975 | 1.221 | 0.128 |
| Jewish | 0.884 | 0.763 | 1.024 | 0.100 | 0.980 | 0.836 | 1.149 | 0.805 |
| Muslim | 1.310 | 1.251 | 1.370 | <0.0001 | 1.191 | 1.099 | 1.291 | <0.0001 |
| Sikh | 1.143 | 1.028 | 1.271 | 0.013 | 1.279 | 1.110 | 1.474 | 0.001 |
| Other | 1.752 | 1.631 | 1.883 | <0.0001 | 1.538 | 1.425 | 1.659 | <0.0001 |
| Prefer not to say | 1.234 | 1.162 | 1.310 | <0.0001 | 1.246 | 1.166 | 1.332 | <0.0001 |
| **Smoking** | | | | | | | | |
| Never smoked | ref |  |  |  | ref |  |  |  |
| Former smoker | 1.176 | 1.149 | 1.204 | <0.0001 | 1.190 | 1.160 | 1.221 | <0.0001 |
| Occasional or regular smoker | 1.169 | 1.130 | 1.210 | <0.0001 | 0.959 | 0.925 | 0.995 | 0.024 |
| **Deaf person who uses sign language** | | | | | | | | |
| No | ref |  |  |  | NA |  |  |  |
| Yes | 3.890 | 3.510 | 4.311 | <0.0001 |  |  |  |  |
| **At least one Long Term Condition (excluding LC)** | | | | | | | | |
| No | ref |  |  |  | ref |  |  |  |
| Yes | 1.642 | 1.606 | 1.680 | <0.0001 | 1.965 | 1.916 | 2.014 | <0.0001 |

## Table S3: Odds of being uncertain about having Long Covid compared to reporting having Long Covid (not sure vs yes) using General Practice Patient Survey data (2023)

|  | **Unadjusted** | | | | **Adjusted** | | | |
| --- | --- | --- | --- | --- | --- | --- | --- | --- |
|  | **Odds ratio** | **95% CI**  **lower** | **95% CI**  **upper** | **p** | **Odds ratio** | **95% CI**  **lower** | **95% CI**  **upper** | **p** |
| **Age** | | | | | | | | |
| 16-24yrs | ref |  |  |  | ref |  |  |  |
| 25-34yrs | 0.927 | 0.852 | 1.008 | 0.077 | 0.876 | 0.793 | 0.967 | 0.009 |
| 35-44yrs | 0.909 | 0.840 | 0.982 | 0.016 | 0.868 | 0.788 | 0.957 | 0.005 |
| 45-54yrs | 0.840 | 0.778 | 0.906 | <0.0001 | 0.850 | 0.774 | 0.935 | 0.001 |
| 55-64yrs | 0.857 | 0.795 | 0.923 | <0.0001 | 0.884 | 0.805 | 0.971 | 0.010 |
| 65-74yrs | 0.950 | 0.880 | 1.025 | 0.185 | 0.882 | 0.796 | 0.977 | 0.016 |
| 75-84yrs | 1.072 | 0.987 | 1.164 | 0.100 | 0.999 | 0.892 | 1.118 | 0.981 |
| 85+yrs | 1.126 | 1.004 | 1.263 | 0.043 | 1.097 | 0.947 | 1.271 | 0.216 |
| **Sex** | | | | | | | | |
| Female | ref |  |  |  | ref |  |  |  |
| Male | 1.129 | 1.100 | 1.160 | <0.0001 | 1.134 | 1.101 | 1.167 | <0.0001 |
| Non-binary | 1.224 | 0.968 | 1.548 | 0.092 | 1.394 | 1.077 | 1.805 | 0.012 |
| Prefer to self-describe | 1.040 | 0.795 | 1.360 | 0.776 | 1.035 | 0.775 | 1.382 | 0.815 |
| Prefer not to say | 1.573 | 1.400 | 1.768 | <0.0001 | 1.285 | 1.126 | 1.466 | <0.0001 |
| **Sexual orientation** | | | | | | | | |
| Heterosexual | ref |  |  |  | ref |  |  |  |
| Gay or lesbian | 0.828 | 0.753 | 0.910 | <0.0001 | 0.854 | 0.773 | 0.943 | 0.002 |
| Bisexual | 0.869 | 0.785 | 0.963 | 0.007 | 0.892 | 0.801 | 0.995 | 0.040 |
| Other | 0.929 | 0.831 | 1.037 | 0.190 | 0.877 | 0.776 | 0.991 | 0.035 |
| I would prefer not to say | 1.356 | 1.284 | 1.432 | <0.0001 | 1.186 | 1.113 | 1.264 | <0.0001 |
| **Gender identity is the same as the sex registered at birth** | | | | | | | | |
| Yes | ref |  |  |  | NA |  |  |  |
| No | 1.061 | 0.915 | 1.229 | 0.433 |  |  |  |  |
| Prefer not to say | 1.463 | 1.313 | 1.631 | <0.0001 |  |  |  |  |
| **Parent** | | | | | | | | |
| No | ref |  |  |  | ref |  |  |  |
| Yes | 0.954 | 0.924 | 0.984 | 0.003 | 0.926 | 0.890 | 0.964 | <0.0001 |
| **Carer** | | | | | | | | |
| No | ref |  |  |  | ref |  |  |  |
| 1-9hrs per week | 0.874 | 0.841 | 0.909 | <0.0001 | 0.916 | 0.879 | 0.954 | <0.0001 |
| 10-19hrs | 0.808 | 0.755 | 0.864 | <0.0001 | 0.822 | 0.766 | 0.883 | <0.0001 |
| 20-34hrs | 0.833 | 0.769 | 0.903 | <0.0001 | 0.829 | 0.761 | 0.902 | <0.0001 |
| 35-49hrs | 0.872 | 0.799 | 0.951 | 0.002 | 0.885 | 0.807 | 0.971 | <0.0001 |
| 50hrs+ | 0.830 | 0.785 | 0.878 | <0.0001 | 0.823 | 0.775 | 0.875 | <0.0001 |
| **Ethnicity** | | | | | | | | |
| White - English, Welsh, Scottish, Northern Irish or British | ref |  |  |  | ref |  |  |  |
| White – Irish | 1.045 | 0.919 | 1.189 | 0.499 | 0.993 | 0.867 | 1.137 | 0.919 |
| White - Gypsy or Irish Traveller | 0.478 | 0.306 | 0.747 | 0.001 | 0.508 | 0.310 | 0.831 | 0.007 |
| White - Roma | 1.471 | 0.985 | 2.197 | 0.059 | 1.354 | 0.880 | 2.082 | 0.168 |
| White - Any other White background | 1.198 | 1.137 | 1.264 | <0.0001 | 1.195 | 1.128 | 1.265 | <0.0001 |
| Mixed or Multiple ethnic groups - White and Black Caribbean | 1.011 | 0.830 | 1.232 | 0.913 | 1.025 | 0.836 | 1.258 | 0.810 |
| Mixed or Multiple ethnic groups - White and Black African | 1.071 | 0.826 | 1.389 | 0.603 | 1.083 | 0.823 | 1.426 | 0.568 |
| Mixed or Multiple ethnic groups - White and Asian | 0.929 | 0.782 | 1.104 | 0.404 | 0.884 | 0.737 | 1.061 | 0.186 |
| Mixed or Multiple ethnic groups - Any other Mixed or multiple ethnic background | 1.067 | 0.919 | 1.240 | 0.394 | 1.102 | 0.941 | 1.291 | 0.226 |
| Asian or Asian British - Indian | 1.365 | 1.278 | 1.458 | <0.0001 | 1.304 | 1.169 | 1.454 | <0.0001 |
| Asian or Asian British – Pakistani | 1.176 | 1.093 | 1.265 | <0.0001 | 1.118 | 0.995 | 1.256 | 0.061 |
| Asian or Asian British - Bangladeshi | 1.291 | 1.141 | 1.460 | <0.0001 | 1.224 | 1.048 | 1.430 | 0.011 |
| Asian or Asian British – Chinese | 1.737 | 1.497 | 2.014 | <0.0001 | 1.752 | 1.495 | 2.053 | <0.0001 |
| Asian or Asian British - Any other Asian background | 1.276 | 1.166 | 1.395 | <0.0001 | 1.266 | 1.140 | 1.405 | <0.0001 |
| Black, Black British, Caribbean or African - Caribbean | 1.126 | 0.989 | 1.281 | 0.072 | 1.169 | 1.019 | 1.340 | 0.026 |
| Black, Black British, Caribbean or African – African | 1.301 | 1.173 | 1.443 | <0.0001 | 1.351 | 1.207 | 1.513 | <0.0001 |
| Black, Black British, Caribbean or African - Any other Black, Black British, Caribbean or African background | 1.209 | 1.019 | 1.435 | 0.030 | 1.172 | 0.979 | 1.404 | 0.084 |
| Other ethnic group - Arab | 1.438 | 1.216 | 1.700 | <0.0001 | 1.461 | 1.203 | 1.775 | <0.0001 |
| Any other ethnic group | 1.156 | 1.056 | 1.267 | 0.002 | 1.126 | 1.014 | 1.250 | 0.026 |
| **Patient IMD quintile** | | | | | | | | |
| 1 (most deprived) | 0.984 | 0.944 | 1.026 | 0.455 | 0.971 | 0.927 | 1.016 | 0.200 |
| 2 | 1.017 | 0.974 | 1.061 | 0.454 | 0.993 | 0.948 | 1.040 | 0.765 |
| 3 | 0.998 | 0.956 | 1.043 | 0.936 | 0.989 | 0.945 | 1.036 | 0.642 |
| 4 | 0.983 | 0.940 | 1.028 | 0.458 | 0.987 | 0.942 | 1.035 | 0.586 |
| 5 (least) | ref |  |  |  | ref |  |  |  |
| **Work status** | | | | | | | | |
| In full-time paid work (30 hours or more each week) | ref |  |  |  | ref |  |  |  |
| In part-time paid work (under 30 hours each week) | 1.019 | 0.979 | 1.062 | 0.351 | 1.085 | 1.039 | 1.133 | <0.0001 |
| In full-time education at school, college or university | 1.091 | 0.991 | 1.202 | 0.077 | 0.917 | 0.816 | 1.031 | 0.146 |
| Unemployed | 1.215 | 1.137 | 1.298 | <0.0001 | 1.181 | 1.100 | 1.268 | <0.0001 |
| Permanently sick or disabled | 0.856 | 0.815 | 0.899 | <0.0001 | 0.937 | 0.887 | 0.990 | 0.020 |
| Fully retired from work | 1.219 | 1.178 | 1.262 | <0.0001 | 1.281 | 1.213 | 1.354 | <0.0001 |
| Looking after the family or home | 1.283 | 1.204 | 1.368 | <0.0001 | 1.391 | 1.297 | 1.491 | <0.0001 |
| Doing something else | 1.244 | 1.153 | 1.343 | <0.0001 | 1.243 | 1.146 | 1.348 | <0.0001 |
| **Religion** | | | | | | | | |
| None | ref |  |  |  | ref |  |  |  |
| Buddhist | 1.061 | 0.918 | 1.225 | 0.425 | 0.919 | 0.783 | 1.079 | 0.301 |
| Christian | 1.061 | 1.030 | 1.094 | <0.0001 | 1.023 | 0.989 | 1.057 | 0.185 |
| Hindu | 1.378 | 1.259 | 1.508 | <0.0001 | 1.082 | 0.949 | 1.234 | 0.238 |
| Jewish | 0.835 | 0.693 | 1.005 | 0.056 | 0.832 | 0.680 | 1.017 | 0.072 |
| Muslim | 1.245 | 1.180 | 1.313 | <0.0001 | 1.036 | 0.943 | 1.139 | 0.461 |
| Sikh | 1.237 | 1.092 | 1.400 | 0.001 | 0.968 | 0.820 | 1.144 | 0.706 |
| Other | 0.902 | 0.827 | 0.985 | 0.021 | 0.890 | 0.811 | 0.978 | 0.015 |
| Prefer not to say | 1.402 | 1.309 | 1.502 | <0.0001 | 1.215 | 1.125 | 1.313 | <0.0001 |
| **Smoking** | | | | | | | | |
| Never smoked | ref |  |  |  | ref |  |  |  |
| Former smoker | 0.995 | 0.967 | 1.024 | 0.721 | 1.053 | 1.020 | 1.086 | 0.001 |
| Occasional or regular smoker | 1.191 | 1.144 | 1.239 | <0.0001 | 1.274 | 1.220 | 1.330 | <0.0001 |
| **Deaf person who uses sign language** | | | | | | | | |
| Yes | ref |  |  |  | NA |  |  |  |
| No | 2.196 | 1.916 | 2.518 | <0.0001 |  |  |  |  |
| **At least one Long Term Condition (excluding LC)** | | | | | | | | |
| No | ref |  |  |  | ref |  |  |  |
| Yes | 0.781 | 0.761 | 0.803 | <0.0001 | 0.764 | 0.741 | 0.788 | <0.0001 |

## Table S4: Odds of being uncertain about having Long Covid compared to not having Long Covid (not sure vs no) using General Practice Patient Survey data (2023)

|  | **Unadjusted** | | | | **Adjusted** | | | |
| --- | --- | --- | --- | --- | --- | --- | --- | --- |
|  | **Odds ratio** | **95% CI**  **lower** | **95% CI**  **upper** | **p** | **Odds ratio** | **95% CI**  **lower** | **95% CI**  **upper** | **p** |
| **Age** | | | | | | | | |
| 16-24yrs | ref |  |  |  | ref |  |  |  |
| 25-34yrs | 1.033 | 0.982 | 1.087 | 0.215 | 0.998 | 0.947 | 1.053 | 0.954 |
| 35-44yrs | 1.208 | 1.152 | 1.267 | <0.0001 | 1.117 | 1.061 | 1.176 | <0.0001 |
| 45-54yrs | 1.237 | 1.182 | 1.296 | <0.0001 | 1.102 | 1.049 | 1.157 | <0.0001 |
| 55-64yrs | 1.103 | 1.055 | 1.154 | <0.0001 | 0.964 | 0.919 | 1.012 | 0.136 |
| 65-74yrs | 0.849 | 0.810 | 0.888 | <0.0001 | 0.719 | 0.685 | 0.756 | <0.0001 |
| 75-84yrs | 0.660 | 0.628 | 0.693 | <0.0001 | 0.549 | 0.521 | 0.580 | <0.0001 |
| 85+yrs | 0.584 | 0.547 | 0.625 | <0.0001 | 0.479 | 0.445 | 0.516 | <0.0001 |
| **Sex** | | | | | | | | |
| Female | ref |  |  |  | ref |  |  |  |
| Male | 0.971 | 0.955 | 0.987 | <0.0001 | 0.972 | 0.956 | 0.989 | 0.002 |
| Non-binary | 1.735 | 1.504 | 2.002 | <0.0001 | 1.282 | 1.102 | 1.493 | 0.001 |
| Prefer to self-describe | 2.076 | 1.742 | 2.474 | <0.0001 | 1.463 | 1.215 | 1.762 | <0.0001 |
| Prefer not to say | 1.887 | 1.767 | 2.015 | <0.0001 | 1.266 | 1.175 | 1.363 | <0.0001 |
| **Sexual orientation** | | | | | | | | |
| Heterosexual |  |  |  |  |  |  |  |  |
| Gay or lesbian | 1.180 | 1.109 | 1.257 | <0.0001 | 1.029 | 0.964 | 1.098 | 0.389 |
| Bisexual | 1.324 | 1.238 | 1.416 | <0.0001 | 1.062 | 0.990 | 1.140 | 0.093 |
| Other | 1.377 | 1.282 | 1.048 | <0.0001 | 1.078 | 0.997 | 1.165 | 0.058 |
| I would prefer not to say | 1.576 | 1.528 | 1.626 | <0.0001 | 1.195 | 1.152 | 1.240 | <0.0001 |
| **Gender identity is the same as the sex registered at birth** | | | | | | | | |
| Yes | ref |  |  |  | NA |  |  |  |
| No | 1.548 | 1.412 | 1.696 | <0.0001 |  |  |  |  |
| Prefer not to say | 2.108 | 1.981 | 2.242 | <0.0001 |  |  |  |  |
| **Parent** | | | | | | | | |
| No | ref |  |  |  | ref |  |  |  |
| Yes | 1.214 | 1.191 | 1.238 | <0.0001 | 0.992 | 0.968 | 1.017 | 0.546 |
| **Carer** | | | | | | | | |
| No | ref |  |  |  | ref |  |  |  |
| 1-9hrs per week | 1.285 | 1.253 | 1.317 | <0.0001 | 1.285 | 1.252 | 1.318 | <0.0001 |
| 10-19hrs | 1.443 | 1.380 | 1.509 | <0.0001 | 1.341 | 1.280 | 1.405 | <0.0001 |
| 20-34hrs | 1.575 | 1.493 | 1.661 | <0.0001 | 1.413 | 1.337 | 1.494 | <0.0001 |
| 35-49hrs | 1.652 | 1.561 | 1.748 | <0.0001 | 1.435 | 1.354 | 1.522 | <0.0001 |
| 50hrs+ | 1.270 | 1.224 | 1.317 | <0.0001 | 1.253 | 1.207 | 1.302 | <0.0001 |
| **Ethnicity** | | | | | | | | |
| White - English, Welsh, Scottish, Northern Irish or British | ref |  |  |  | ref |  |  |  |
| White – Irish | 1.065 | 0.983 | 1.153 | 0.123 | 1.031 | 0.948 | 1.121 | 0.478 |
| White - Gypsy or Irish Traveller | 1.828 | 1.283 | 2.606 | 0.001 | 1.255 | 0.858 | 1.835 | 0.242 |
| White - Roma | 1.812 | 1.443 | 2.276 | <0.0001 | 1.467 | 1.154 | 1.866 | 0.002 |
| White - Any other White background | 1.459 | 1.413 | 1.507 | <0.0001 | 1.296 | 1.252 | 1.341 | <0.0001 |
| Mixed or Multiple ethnic groups - White and Black Caribbean | 1.299 | 1.147 | 1.472 | <0.0001 | 1.061 | 0.932 | 1.208 | 0.369 |
| Mixed or Multiple ethnic groups - White and Black African | 1.388 | 1.181 | 1.632 | <0.0001 | 1.123 | 0.947 | 1.332 | 0.182 |
| Mixed or Multiple ethnic groups - White and Asian | 1.342 | 1.199 | 1.501 | <0.0001 | 1.118 | 0.992 | 1.260 | 0.068 |
| Mixed or Multiple ethnic groups - Any other Mixed or multiple ethnic background | 1.693 | 1.540 | 1.861 | <0.0001 | 1.407 | 1.275 | 1.552 | <0.0001 |
| Asian or Asian British - Indian | 1.431 | 1.378 | 1.487 | <0.0001 | 1.248 | 1.170 | 1.330 | <0.0001 |
| Asian or Asian British – Pakistani | 1.790 | 1.711 | 1.872 | <0.0001 | 1.250 | 1.165 | 1.342 | <0.0001 |
| Asian or Asian British - Bangladeshi | 1.940 | 1.802 | 2.089 | <0.0001 | 1.320 | 1.203 | 1.449 | <0.0001 |
| Asian or Asian British – Chinese | 1.553 | 1.435 | 1.680 | <0.0001 | 1.579 | 1.454 | 1.716 | <0.0001 |
| Asian or Asian British - Any other Asian background | 1.586 | 1.504 | 1.673 | <0.0001 | 1.354 | 1.273 | 1.440 | <0.0001 |
| Black, Black British, Caribbean or African - Caribbean | 1.036 | 0.958 | 1.120 | 0.377 | 0.873 | 0.804 | 0.948 | 0.001 |
| Black, Black British, Caribbean or African – African | 0.774 | 0.729 | 0.821 | <0.0001 | 0.639 | 0.600 | 0.682 | <0.0001 |
| Black, Black British, Caribbean or African - Any other Black, Black British, Caribbean or African background | 1.104 | 0.998 | 1.222 | 0.055 | 0.889 | 0.799 | 0.990 | 0.032 |
| Other ethnic group - Arab | 1.701 | 1.546 | 1.872 | <0.0001 | 1.285 | 1.151 | 1.434 | <0.0001 |
| Any other ethnic group | 1.659 | 1.568 | 1.754 | <0.0001 | 1.310 | 1.230 | 1.396 | <0.0001 |
| **Patient IMD quintile** | | | | | | | | |
| 1 (most deprived) | 1.661 | 1.618 | 1.704 | <0.0001 | 1.388 | 1.349 | 1.427 | <0.0001 |
| 2 | 1.419 | 1.382 | 1.457 | <0.0001 | 1.250 | 1.216 | 1.286 | <0.0001 |
| 3 | 1.252 | 1.219 | 1.286 | <0.0001 | 1.166 | 1.134 | 1.199 | <0.0001 |
| 4 | 1.120 | 1.090 | 1.151 | <0.0001 | 1.083 | 1.053 | 1.115 | <0.0001 |
| 5 (least) | ref |  |  |  | ref |  |  |  |
| **Work status** | | | | | | | | |
| In full-time paid work (30 hours or more each week) | ref |  |  |  | NA |  |  |  |
| In part-time paid work (under 30 hours each week) | 1.014 | 0.988 | 1.040 | 0.293 |  |  |  |  |
| In full-time education at school, college or university | 0.878 | 0.827 | 0.931 | <0.0001 |  |  |  |  |
| Unemployed | 1.361 | 1.308 | 1.417 | <0.0001 |  |  |  |  |
| Permanently sick or disabled | 1.643 | 1.589 | 1.699 | <0.0001 |  |  |  |  |
| Fully retired from work | 0.658 | 0.645 | 0.672 | <0.0001 |  |  |  |  |
| Looking after the family or home | 1.125 | 1.083 | 1.168 | <0.0001 |  |  |  |  |
| Doing something else | 1.152 | 1.100 | 1.206 | <0.0001 |  |  |  |  |
| **Religion** | | | | | | | | |
| None | ref |  |  |  | ref |  |  |  |
| Buddhist | 1.371 | 1.252 | 1.501 | <0.0001 | 1.121 | 1.015 | 1.238 | 0.024 |
| Christian | 0.969 | 0.951 | 0.988 | 0.001 | 1.076 | 1.054 | 1.098 | <0.0001 |
| Hindu | 1.336 | 1.268 | 1.408 | <0.0001 | 1.153 | 1.067 | 1.244 | <0.0001 |
| Jewish | 0.738 | 0.653 | 0.834 | <0.0001 | 0.826 | 0.727 | 0.939 | 0.004 |
| Muslim | 1.630 | 1.578 | 1.684 | <0.0001 | 1.245 | 1.176 | 1.317 | <0.0001 |
| Sikh | 1.413 | 1.312 | 1.523 | <0.0001 | 1.217 | 1.103 | 1.343 | <0.0001 |
| Other | 1.582 | 1.492 | 1.676 | <0.0001 | 1.377 | 1.295 | 1.463 | <0.0001 |
| Prefer not to say | 1.730 | 1.662 | 1.801 | <0.0001 | 1.517 | 1.449 | 1.587 | <0.0001 |
| **Smoking** | | | | | | | | |
| Never smoked | ref |  |  |  | ref |  |  |  |
| Former smoker | 1.170 | 1.150 | 1.191 | <0.0001 | 1.257 | 1.232 | 1.281 | <0.0001 |
| Occasional or regular smoker | 1.392 | 1.359 | 1.426 | <0.0001 | 1.218 | 1.187 | 1.250 | <0.0001 |
| **Deaf person who uses sign language** | | | | | | | | |
| Yes | ref |  |  |  | NA |  |  |  |
| No | 0.565 | 0.507 | 0.629 | <0.0001 |  |  |  |  |
| **At least one Long Term Condition (excluding LC)** | | | | | | | | |
| No | ref |  |  |  | ref |  |  |  |
| Yes | 1.283 | 1.263 | 1.304 | <0.0001 | 1.485 | 1.458 | 1.512 | <0.0001 |
